# m^6^A demethylase FTO drives pancreatic ductal adenocarcinoma tumorigenesis and metastasis through remodeling PFKM mediated glycolysis

**DOI:** 10.1038/s41419-025-08049-2

**Published:** 2025-11-03

**Authors:** Zhen Tan, Jianhui Yang, Yueyue Chen, Heng Zhu, Xiaomeng Liu, He Xu, Qingcai Meng, Mingming Xiao, Rong Tang, Zeyin Rong, Xianjun Yu, Chen Liang, Jin Xu

**Affiliations:** 1 Department of Pancreatic Surgery, Shanghai Cancer Center, Shanghai, China; 2 Department of Oncology, Shanghai Medical College, Shanghai, China; 3 Shanghai Pancreatic Cancer Institute, Shanghai, China; 4 Shanghai Key Laboratory of Precision Medicine for Pancreatic Cancer, Shanghai, China; 5 Pancreatic Cancer Institute, Shanghai, China; 6 Department of Hepatopancreatobiliary Surgery, The Affiliated Hospital of Qingdao University, Qingdao, China

**Keywords:** Metastasis, Cancer therapy

## Abstract

N6-methyladenosine (m^6^A) modification has emerged as a common chemical modification in epigenetic regulation. However, whether this m^6^A modification is involved in glycolysis metabolism in pancreatic ductal adenocarcinoma (PDAC) remains elusive. Multiomics integration strategies, including metabolomics, m^6^A-seq and transcriptome sequencing, were utilized to evaluate the associations between m^6^A modifications and key processes of glucose metabolism in PDAC. Spontaneous PDAC mice (LSLKras^G12D/+^, LSL-Trp53^R172H/+^, Pdx1-Cre; KPC) with FTO-conditional knockout and organoids were used to evaluate the effects of FTO stimulation on PDAC cell glycolysis and tumorigenesis. Series of in vivo and vitro functional analysis revealed that FTO promoted migratory capacity and glycolysis of PDAC cells. Mechanistically, FTO elevates the mRNA expression of the transcription factor C-Jun in a m^6^A-YTHDF2-dependent manner and further transcriptionally upregulates PFKM expression. Translational studies involving organoid models and xenograft tumor models revealed that the use of FTO inhibitors significantly suppressed PDAC growth. Our findings uncover that targeting the m^6^A-dependent FTO/C-Jun/PFKM glycolysis regulatory axis may be essential for the prevention and treatment of PDAC.

## Introduction

Pancreatic ductal adenocarcinoma (PDAC) is a solid malignancy with the poorest prognosis [1]. PDAC was the seventh leading cause of cancer death worldwide in 2018. The 5-year patient survival rate at the time of diagnosis is approximately 10% [2]. PDAC has a complex landscape of germline or acquired genetic mutations, including mutations in TP53, KRAS, SMAD4, and CDKN2A. The complex diverse tumor microenvironment is generally considered a major factor in resistance to radiation, chemotherapy, and molecular targeted therapies [3, 4].

N6-methyladenosine (m^6^A) is the most abundant posttranscriptional modification within mRNAs and regulates various biological processes involved in tumor progression [5]. In mammals, m^6^A methylation processes are dynamic and post-transcriptionally regulated by “writers”, “erasers” and “readers” [6]. Recent studies have shown that m^6^A participates in metabolism and regulates tumor initiation or progression. The Warburg effect, a hallmark of invasive cancer metabolism, accelerates glycolysis activation in PDAC partially through remodeling of the extracellular matrix [7, 8]. Higher glycolysis in PDAC is considered an adaptation to the hypoxic microenvironment, thus providing continuous energy to sustain tumor cell invasion and migration. A growing body of literature has revealed that m^6^A methylation is involved in tumorigenesis via dynamic mRNA transcript alterations. Liu et al. demonstrated that fat mass and obesity-associated protein (FTO) stimulates the glycolytic activity of tumor cells, thus modulating CD8^+^ T-cell function and promoting tumor growth [9]. In colorectal cancer, METTL3 regulates HK2 and SLC2A1 stabilization via m^6^A modification, illustrating intersectional effects with epitranscriptomic changes and glycolysis in carcinogenesis [10]. However, the molecular basis for glycolysis and reprogramming of the RNA epitranscriptome in pancreatic cancer remains unclear.

Here, we investigated the role of FTO in determining global m^6^A levels in PDAC and investigated its oncogenic role in metabolism and tumorigenesis. Our findings show that FTO dominates glycolysis by preserving C-Jun expression in a m^6^A-YTHDF2-dependent manner. Attenuation of FTO expression decreases C-Jun accumulation and subsequently reduces PFKM transcription in PDAC cells, which subsequently abolishes the metabolic barrier to migration. The results of the present study provide new insight into changes in m^6^A modifications in PDAC carcinogenesis and glycolysis.

## Methods

### Patient specimens

We used three cohorts of patients with PDAC who underwent surgery between 2012 and 2019. Cohorts #1 (16 cases of fresh tissues), #2 (18 cases of fresh tissues) and #3 (278 paraffin-embedded tissues) were obtained from the Fudan University Shanghai Cancer Center (FUSCC). None of the patients included in our study received any anticancer treatment, including chemotherapy and radiotherapy, before surgical resection. All patients received strict postoperative follow-up care. Detailed patient information is provided in Supplementary Tables 1–3. All experiments received approval from the Clinical Research Ethics Committee of FUSCC (Project identification code: 2410-Exp085).

### Cell culture and treatment

The human PDAC cell lines MiaPaCa-2, PANC-1, CFPAC-1, and HEK-293T were purchased from American Type Culture Collection (ATCC). CFPAC-1 cells were cultured in IMDM, and other pancreatic ductal cancer cell lines were cultured in DMEM. The media were supplemented with 10% fetal bovine serum (FBS) and 100 U/mL penicillin, and MiaPaCa-2 and PANC-1 cells were grown in a humidified incubator at 37 °C with 5% CO_2_ and assessed for mycoplasma contamination via PCR every 3 months. The FTO inhibitors FB23-2 and 2-deoxy-D-glucose (2-DG) were obtained from Selleck (Houston, TX, USA).

### Bioinformatics analysis

PDAC transcriptome profiles with clinical data were obtained from The Cancer Genome Atlas (TCGA, https://portal.gdc.cancer.gov) and Genotype-Tissue Expression (GTEx) databases (http://www.gtexportal.org).

### Total RNA extraction and real-time PCR

Total RNA was extracted from tumor samples and human PDAC cells with TRIzol reagent (Invitrogen, Carlsbad, CA, USA) and subsequently reverse transcribed into cDNA with the Prime Script RT Reagent Kit (Takara, Shanghai, China) according to the manufacturer’s instructions. The 2^-ΔΔCt^ method was used to quantify the mRNA expression levels, and β-actin was used as an internal control.

### Western blot (WB), immunofluorescence (IF), immunohistochemistry (IHC) and enzyme-linked immunosorbent assay (ELISA) procedures

WB, IF, and IHC were performed following standard procedures previously described [11]. We performed ELISA using the cell supernatant according to the manufacturers’ instructions for the Human Phospho-C-Jun (Ser63) and Total C-Jun ELISA Kits (RayBio, PEL-JUN-S63-T). The antibodies used in the present study included those against FTO (1:1000; Abcam), GAPDH (1:20000; Proteintech), E-cadherin (1:20000; Abcam), N-cadherin (1:2000; Abcam), ZO-1 (1:1000; Cell Signaling Technology), PFKM (1:1000; Proteintech), C-Jun (1:1000; Cell Signaling Technology), and YTHDF2 (1:1000; Proteintech). The grayscale of the indicated protein was quantified using image analysis software (ImageJ 1.51e, NIH Image). IF analysis was performed with the following corresponding antibodies: IF: FTO (1:500, Proteintech), C-Jun (1:200, Proteintech), PFKM (1:200, Proteintech), Ki-67 (1:100, Proteintech), and CK19 (1:100, Proteintech). Changes in IF signals were assessed via confocal microscopy (Leica SP5, Leica Biosystems, USA).

### Liquid chromatography‒tandem mass spectrometry (LC‒MS/MS) for m^6^A quantification in mRNAs

A mixture of buffer, S1 nuclease, alkaline phosphatase, and phosphodiesterase I was added to 1 μg of RNA and then incubated at 37°C. The nucleosides were extracted with chloroform after the RNA was completely digested. Liquid chromatography–electrospray ionization tandem mass spectrometry was used to analyze the resulting aqueous layer. The m^6^A/A ratio in mRNA was calculated on the basis of the standard curve derived from pure nucleoside standards.

### Seahorse metabolic analysis

The extracellular acidification rate (ECAR) was measured via the Seahorse XF Glycolysis Stress Test Kit and the Cell Mito Stress Test Kit in a Bioscience XF96 Extracellular Flux Analyzer. MiaPaCa-2 and PANC-1 cells were seeded into 96-well cell culture plates in DMEM supplemented with 10% FBS and incubated at 37 °C overnight. After the baseline concentration was measured, glucose, oligomycin, and 2-DG were sequentially added to each well for ECAR measurement. Seahorse XF96 Wave software was used to analyze the data after normalization based on cell number.

### m^6^A dot blot assay

In summary, RNA samples were transferred onto Amersham Hybond-N+ membranes with a Bio-Dot Apparatus and then exposed to UV cross-linking. The membranes were stained with 0.02% methylene blue and scanned to evaluate the total RNA content. The membranes were rinsed with phosphate-buffered saline supplemented with 0.1% Tween 20, and subsequently blocked using a 5% non-fat milk solution. This was followed by an overnight incubation at 4 °C with an anti-m^6^A antibody. Detection was achieved using a horseradish peroxidase-conjugated secondary antibody.

### RNA immunoprecipitation and SELECT detection assay

An RNA immunoprecipitation (RIP) assay was conducted using a Magna RIP Kit (Millipore, Billerica, MA, USA) following the manufacturer’s instructions. In brief, an anti-YTHDF2 antibody or negative control immunoglobulin G (IgG) was used for the RIP assay. Transfected cells were prepared using RIP lysis buffer, and the RNA‒protein complexes were immunoprecipitated via an appropriate antibody. The coprecipitated RNAs were purified via phenol: chloroform: isoamyl alcohol and subjected to agarose gel electrophoresis or qRT‒PCR analysis. The SELECT assay was performed with an Epi-SELECT m^6^A site identification kit (Guangzhou Epibiotek Co., Ltd.) as previously described [12].

### In vitro cell behavior assays

Cell invasion and migration assays were performed using transwell chambers. A total of 8 × 10^4^ cells per well were seeded into the upper chambers and cultured in 200 μL of serum-free medium. The lower chamber was filled with 800 μL of medium containing 10% FBS as a nutritional attractant. After incubation for 24 h, the lower surface of the plates containing cells was fixed with 4% polyformaldehyde and imaged after staining with 0.2% crystal violet for 30 min. Finally, the migrated or invaded cells were quantified by capturing three randomly chosen microscopic fields for each sample.

For the wound healing assay, 6–8 × 10^5^ cells were seeded into 6-well plates and cultured until 90% confluence. The cell monolayer was subsequently gently scratched with a 200-μL pipette tip, washed twice with PBS and incubated without FBS medium. The areas of the wound-like gaps were photographed at 0 h and 48 h. Experiments were performed in triplicate for each group.

### Generation of transgenic mice

LSL-Kras^G12D/+^, LSL-Trp53^R172H/+^, and FTO^flox/flox^ mice were purchased from GemPharmatech (Nanjing, China), and B6-Pdx1-iCre mice were purchased from The Jackson Laboratory. These mice were interbred to generate LSL-Kras^G12D/+^, LSL-Trp53^R172H/+^, Pdx1-Cre (KPC), and FTO^flox/flox^ KPC (FTO/KPC, FKPC) strains. All animal experiments were approved and performed according to protocols approved by the Research Ethics Committee of FUSCC (Project identification code: FUSCC-IACUC-2024334). All the mice were maintained at the Shanghai Cancer Center-Specific Pathogen Free (SPF) facility.

### In vivo xenograft model

To illustrate the effect of FTO on tumors in vivo, 4-week-old female BALB/c nude mice were purchased from Shanghai SLAC Laboratory. While the choice of female mice minimizes immune rejection and behavioral confounders, it does not address potential sex-specific differences in tumor biology or drug response. Future investigations directly comparing both sexes are warranted. To construct the PDAC xenograft model, 2 × 10^6^ KPC cells were injected into the right flank of the mice subcutaneously. For small molecule inhibitor treatment, 2 mg/kg FB23-2 or control solvent was delivered via intraperitoneal injection every day for 2 weeks after tumor inoculation. The tumor volume was assessed using an external caliper once per week and calculated as follows: tumor volume = (longer diameter × shorter diameter^2^)/2. The mice were euthanized at the 5th week, the tumors were surgically dissected for IHC examination, and the tumor weights were also measured. The model of liver metastases was established by suspending 5 × 10^5^ KPC/FKPC cells into the subcapsule of the spleen of C57BL/6 mice (4–5 weeks old), and the abdominal cavity was closed after compression hemostasis. H&E staining confirmed and quantified the liver metastatic nodules. All animal experiments complied with the protocols and were approved by the Research Ethics Committee of FUSCC.

### Organoid generation

Freshly isolated mouse pancreatic adenocarcinoma tissue was cut into small pieces, treated with Tumor Tissue Digestion Solution (bioGenousTM, #K601003), placed in a 37 °C shaker and gently rotated for 1 h. Then, the cell suspension was subsequently filtered through a 100 μm cell strainer (Corning), centrifuged at 350 × *g* for 5 min to remove the supernatant, resuspended in Red Blood Cell Lysis Solution (bioGenousTM, #E238010) and lysed at 4 °C for 5 min. Afterward, the supernatant was removed by centrifugation at 400 × *g* for 5 min, and the precipitate was resuspended in a mixture of Pancreatic Cancer Organoid Basal Medium (bioGenousTM, #K2101-PC) and Organoid Culture Matrigel (bioGenousTM, #M315066) (3:7 ratio). Approximately 30 µl of Matrigel-containing organoids was added to the bottom center of a 48-well cell culture plate (Corning), which was then inverted and placed in a cell culture incubator for 20 min to allow the Matrigel to solidify. Then, 400 µl of pancreatic cancer organoid basal medium was added to establish MDOs. Every 1–2 weeks, the cells were passaged at a 1:3 ratio. The viability of organoid tissues was assessed using a CellTiter-Glo kit (Promega, #G9683).

### Measurement of ATP levels

ATP levels were measured using an ATP determination kit following the manufacturer’s instructions. In brief, ATP was extracted from 1 × 10^6^ cells by mixing them with 2.5% trichloroacetic acid (TCA). To calculate the ATP concentration in each sample, the ATP standard was continuously diluted to obtain a standard curve. The relative ATP concentration was subsequently measured and normalized to that of the control group. All the experiments were performed in triplicate.

### Metabolomic analysis and transcriptome sequencing

For metabolomics analysis, 50 mg of PDAC samples and 500 µL of precooled extractant (70% methanol aqueous solution) were placed in a 2-mL EP tube. After homogenization and centrifugation, 200 µL of the supernatant was removed for LC‒MS/MS system analysis. The R package “MetaboAnalystR” was applied for OPLS-discriminant analysis (OPLS-DA). Variable importance in projection (VIP) ≥ 1 and absolute log fold change (logFC) ≥ 1.5 served as cutoffs for selecting significantly different metabolites between groups.

For transcriptome sequencing, after quantification and qualification, 1 µg of RNA from each sample was used as input for the RNA sample preparations. The NEBNext® UltraTM RNA Library Prep Kit for Illumina® (NEB, USA) was used to generate the sequencing libraries according to the manufacturer’s recommendations, and index codes were added to attribute sequences. The Illumina HiSeq platform was used to sequence the library preparations after cluster generation. Differentially expressed genes (DEGs) between the two groups were obtained via DESeq2 v1.22.1 analysis, and the *P* value was corrected via the Benjamini and Hochberg method. A corrected *P* < 0.05 and |logFC | > 1.5 were used as the thresholds for the significant difference standard.

### Chromatin immunoprecipitation (ChIP) assay

ChIP was performed according to the instructions of the SimpleChIP plus Enzymatic Chromatin IP Kit (Cell Signaling Technology). In brief, the indicated cells were grown to 80–90% confluence and subsequently collected and crosslinked with 1% formaldehyde. After centrifugation, the cell pellets were sonicated, and protein G magnetic beads were precleared with chromatin solution. The clarified nuclear extract fraction was incubated with an anti-C-Jun antibody or anti-IgG antibody. PCR amplification was performed to determine whether C-Jun binds to PFKM promoter genomic regions. The PCR amplicon products were visualized via 2% agarose gel electrophoresis with ethidium bromide.

### Luciferase assay

The PFKM promoter region and the two indicated mutant site fragments were cloned upstream of the pGL3-basic luciferase vector. HEK-293T cells were seeded in a 12-well plate, and the resulting dual-luciferase reporter plasmids (WT or Mut) were transfected with plasmids expressing C-Jun and Renilla plasmids via Lipofectamine 3000. A dual-luciferase reporter assay kit (Vazyme, China) was used to measure luciferase activity, which was normalized against Renilla activity, using a plate reader.

### Statistical analysis

GraphPad Prism 8.0 and SPSS 23.0 were used for statistical analysis and graphing. Student’s *t* test or one-way ANOVA was used to evaluate the data. Correlations between FTO and PFKM expression were evaluated via Pearson correlation analysis. Survival was evaluated via the Kaplan‒Meier method followed by the log-rank test. Statistical differences were considered significant at *P < 0.05, **P < 0.01, and ***P < 0.001. The results are presented as the means ± SDs of at least three independent experiments.

## Results

### m^6^A levels in PDAC are determined by FTO

**Fig. 1:**
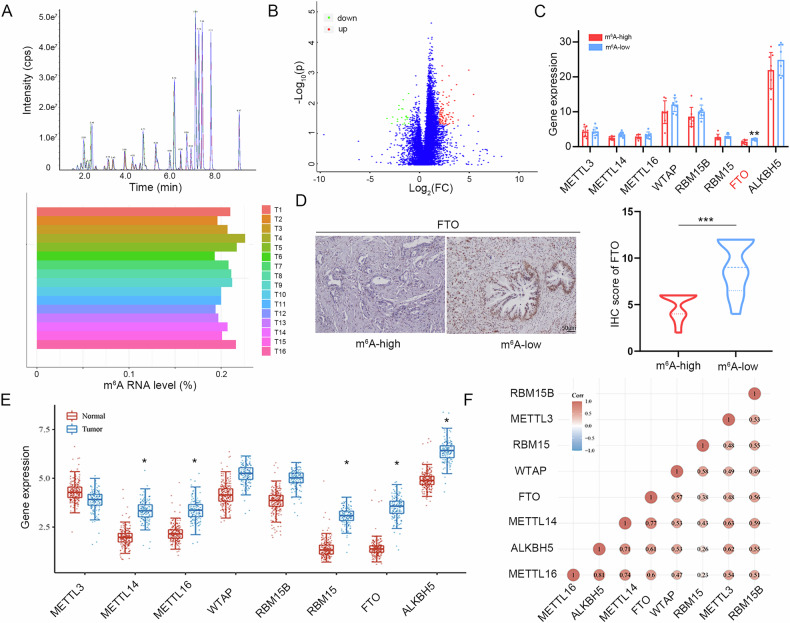
Global m^6^A is modulated by FTO in PDAC. **A** Global m^6^A levels were measured using LC‒MS/MS in tumor tissues from Cohort #1 PDAC patients. **B** RNA-seq experiments were performed to gain insight into the mode of action of the m^6^A mechanism. Volcano plot showing the differentially expressed genes (fold change > 1.5, *P* < 0.05). **C** Expression levels of m^6^A regulators in m^6^A-high and m^6^A-low subgroup patients. **D** Representative IHC images of FTO in the m^6^A-high and m^6^A-low subgroups from Cohort #1. IHC staining was also scored and analyzed. **E** Boxplot of 8 m^6^A-related methyltransferases and demethylases in PDAC tissues and normal tissues in TCGA database. **F** Expression correlation between m^6^A regulators in TCGA-PDAC cohort.

To investigate the role of m^6^A modification in PDAC, global m^6^A levels were examined in 16 tumor samples using LC‒MS/MS (Fig. 1A). According to the ratio of m^6^A levels, the PDAC samples were divided into a m^6^A-high subgroup and a m^6^A-low subgroup (Cohort #1). Global m^6^A levels are determined by writers and erasers. RNA-seq was applied to explore the differences in m^6^A modulator expression levels between the m^6^A-high and m^6^A-low subgroups. DEGs were obtained and are shown in a volcano plot (Fig. 1B). Among the major components of the writers and erasers, FTO was downregulated in the m^6^A-high subgroup (Fig. 1C). To validate the transcriptome sequencing results, we examined FTO protein expression in the two subgroups using IHC. This result was consistent with the RNA-seq results, and FTO was downregulated in the m^6^A-high subgroup (Fig. 1D). Furthermore, we evaluated the distinct expression levels of 8 m^6^A-associated genes between tumor and normal tissues in TCGA-PDAC dataset. METTL14, METTL16, RBM15, FTO and ALKBH5 were significantly overexpressed in PDAC samples (Fig. 1E). In addition, as shown in Fig. 1F, FTO was positively correlated with other m^6^A methylation regulators.

### FTO facilitates PDAC cell migration and invasion in vitro and in vivo

**Fig. 2:**
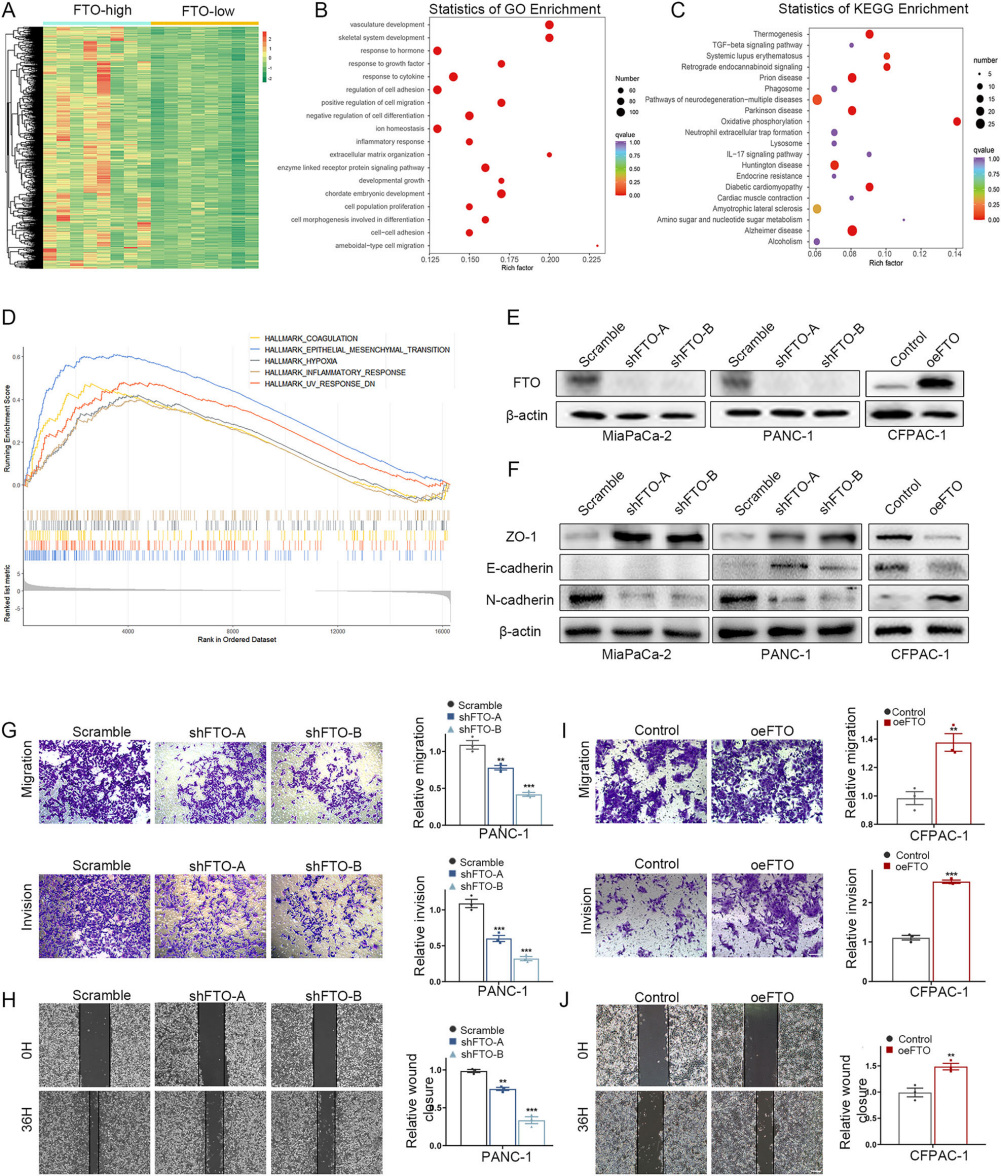
FTO promoted pancreatic cancer cell migration and invasion. **A** Heatmap showing the significant DEGs in the transcriptome data between the FTO-high subgroup samples and the FTO-low subgroup samples. **B**, **C** GO and KEGG analyses revealed enrichment of processes related to cell migration, the regulation of cell adhesion and the TGF-beta signaling pathway. **D** GSEA pathway enrichment analysis results. **E** FTO knockdown and overexpression were verified at the protein level. **F** Western blot analysis was used to detect epithelial and mesenchymal cell phenotype marker levels. **G**–**J** Transwell and wound healing assays revealed the suppressed migratory capacity of PDAC cells following FTO knockdown. In contrast, FTO overexpression promoted PDAC cell migration and invasion.

To determine the role of FTO in human PDAC, we implemented a bioinformatics analysis of transcriptome data regrouped based on the FTO expression level (Fig. 2A). Functional enrichment and pathway analyses were subsequently performed. Among these differentially expressed genes (DEGs), Kyoto Encyclopedia of Genes and Genomes (KEGG) enrichment analyses revealed that the TGF-beta signaling pathway was related to FTO-dependent transcription. Positive regulation of cell migration, regulation of cell adhesion and cell population proliferation were the most enriched terms in the Gene Ontology (GO) enrichment analysis (Fig. 2B, C). Moreover, epithelial‒mesenchymal transition (EMT)-related pathways were identified via gene set enrichment analysis (Fig. 2D). Furthermore, FTO protein expression was upregulated in 18 paired PDAC tissue samples via Western blotting (Cohort #2) (Supplementary Fig. S1A). Taken together, these findings indicate that FTO is upregulated in PDAC and may regulate PDAC progression through m^6^A modification. The functional enrichment of genes related to cell migration, EMT and the TGF-beta signaling pathway was directly related to metastasis in PDAC. We thus hypothesized that FTO expression is associated with the invasive phenotypes in PDAC cells. Based on this finding, we stably transfected MiaPaCa-2 and PANC-1 cells with an FTO-specific shRNA and established stable CFPAC-1 cells that ectopically overexpressed FTO (Fig. 2E). To evaluate phenotypic alterations consistent with EMT, we observed that the levels of epithelial markers E-cadherin and ZO-1 were increased, whereas the level of the mesenchymal marker N-cadherin was decreased in FTO-knockdown MiaPaCa-2 and PANC-1 cells. The FTO-overexpressing CFPAC-1 cells also exhibited an EMT phenotype (Fig. 2F). Both wound healing and transwell assays were used to investigate the effects of FTO on PDAC cell migration. Our preliminary data revealed that FTO knockdown markedly reduced migration and invasion abilities (Fig. 2G, H and Supplementary Fig. S1C–D). In contrast, FTO overexpression increased this trend (Fig. 2I, J).

**Fig. 3:**
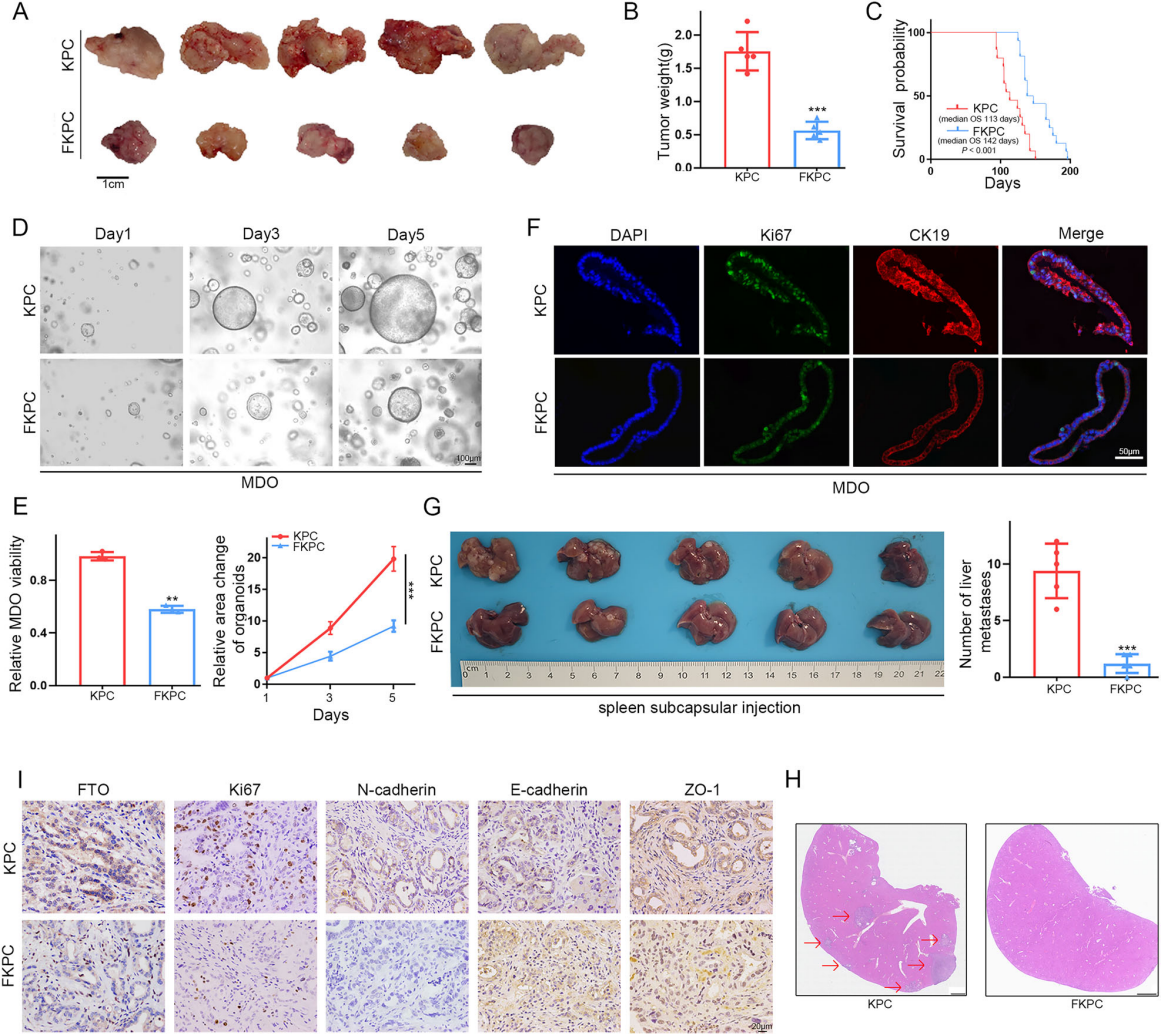
The roles of FTO in PDAC tumorigenesis. **A**, **B** Representative images and tumor weights of isolated KPC/FKPC mouse tumors. **C** Survival analysis of KPC and FKPC mice. *P* values were determined using the log-rank test. **D** Representative brightfield images showing the MDO morphology. **E** The relative viability and volume of KPC/FKPC mouse spontaneous tumor-derived organoids were measured. **F** Immunofluorescence images of CK19, Ki-67 and DAPI staining in MDO. **G** Different primary KPC tumor cell lines were intrasplenically injected into C57BL/6 mice. At the end of the experiment, liver metastases were photographed and dissected to assess the number of metastatic lesions. H&E staining (**H**) and IHC staining (**I**) of FTO, Ki-67 and EMT-related markers in a metastatic model generated with KPC/FKPC cells were performed.

Because FTO loss mediates the reversal of EMT and hence impairs PDAC cell motility and invasiveness, we used autochthonous LSL-Kras^G12D/+^; Trp53^R172H/+^; Pdx1-Cre (KPC) pancreatic tumor-bearing mice and produced FKPC mice by introducing the FTO^flox/flox^ allele. Importantly, compared with KPC mice, FKPC mice presented smaller tumor volumes and lower tumor weights but superior survival outcomes (median OS, 123 vs. 94 days) (Fig. 3A–C). To further investigate the effect of FTO on tumorigenicity, we generated mouse-derived organoids (MDOs) from KPC and FKPC mice. Immunofluorescence, organoid morphology and viability revealed that the organoids and the corresponding primary tumors presented similar oncological features (Fig. 3D–F). To further validate the effect of FTO on PDAC cell metastasis in vivo, we generated a hepatic metastatic xenograft model via intrasplenic injection of KPC or FKPC primary tumor cells. Consistent with the in vitro analyses, H&E staining of excised liver sections confirmed that the loss of FTO expression reduced the number of metastatic hepatic foci (Fig. 3G, H). Similar results were obtained for EMT indicators via IHC validation (Fig. 3I). Collectively, these results indicate that FTO deficiency inhibits PDAC metastasis in vitro and in vivo.

### FTO drives glycolytic metabolism in pancreatic cancer

**Fig. 4:**
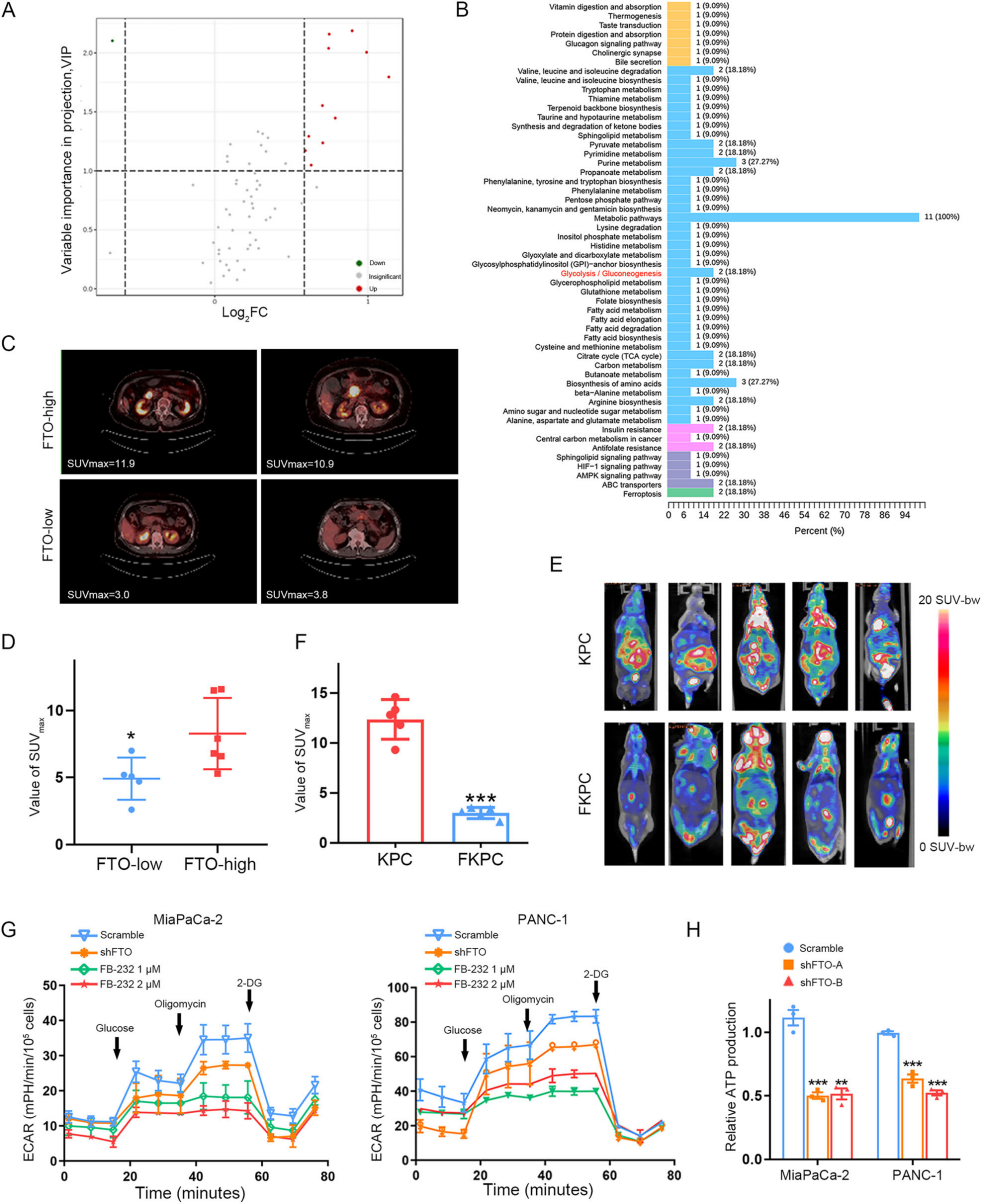
FTO induced glycolytic metabolism in PDAC. **A** Volcano plot analysis of differentially abundant metabolites in FTO-high and FTO-low subgroup tissues via LC‒MS/MS metabolomics. **B** Overview of pathway analysis depicting altered metabolic pathways. **C**, **D** Representative PET/CT images and statistical analysis of SUVmax (FDG maximal standardized uptake value) values between the FTO-high subgroup and low subgroup (Cohort #1). **E**, **F** Representative PET/CT images and statistical analysis of SUVmax values between KPC and FKPC mice. **G** The extracellular acidification rate (ECAR) was detected in MiaPaCa-2 and PANC-1 WT, FTO-knockdown and PDAC cells treated with the indicated concentrations of the FTO inhibitor. **H** FTO knockdown decreased ATP production.

In the volcano plot, the right side shows 11 upregulated metabolites between the FTO-high and low groups, and 1 downregulated metabolite is shown on the left side (Fig. 4A). The significantly changed metabolites are shown separately in the Supplementary Fig. S2. The KEGG Compound database (http://www.kegg.jp/kegg/compound/) and KEGG Pathway database (http://www.kegg.jp/kegg/pathway.html) were used to annotate identified metabolites. The relevant metabolic pathways are represented by a colored histogram within the diagram. Metabolic pathways and glycolysis/gluconeogenesis were enriched in multiple differentially abundant metabolites (Fig. 4B). The key components of the glycolysis pathway, such as fructose 1,6-bisphosphate and acetyl-CoA, were significantly upregulated in the FTO-high subgroup, as shown by sequencing (Supplementary Fig. S2). To investigate the relationship between m^6^A modifications and glucose metabolism in PDAC, we obtained FDG uptake in PDAC patients (cohort #1) via the maximum standardized uptake value (SUVmax). As presented in Fig. 4C, SUVmax indicated relatively higher in the FTO-high subgroup. These data imply that FTO potentially mediates glucose metabolism and carcinogenesis in PDAC. Moreover, we performed FDG-PET on our transgenic mice including KPC and FKPC. The outcomes revealed that the tumors that were null for FTO had significantly less FDG uptake than the KPC tumors did (Fig. 4E, F). To elucidate whether alterations in FTO could influence glycolytic metabolism, the extracellular acidification rate (ECAR) was determined after FTO expression, and activity levels were manipulated. FB23-2, an inhibitor that directly binds to FTO and inhibits its m^6^A demethylase activity, has been identified [13, 14]. Compared with the control, knockdown or various concentrations of FB23-2 significantly reduced the ECAR in MiaPaCa-2 and PANC-1 cells (Fig. 4G). Further functional validation revealed that energy metabolism/ATP production was significantly decreased after FTO knockdown in MiaPaCa-2 and PANC-1 cells (Fig. 4H). These data revealed reduced glucose uptake in PDAC cells deficient in FTO activity.

### RNA‑seq and metabolomics identified PFKM as a downstream effector gene of FTO

**Fig. 5:**
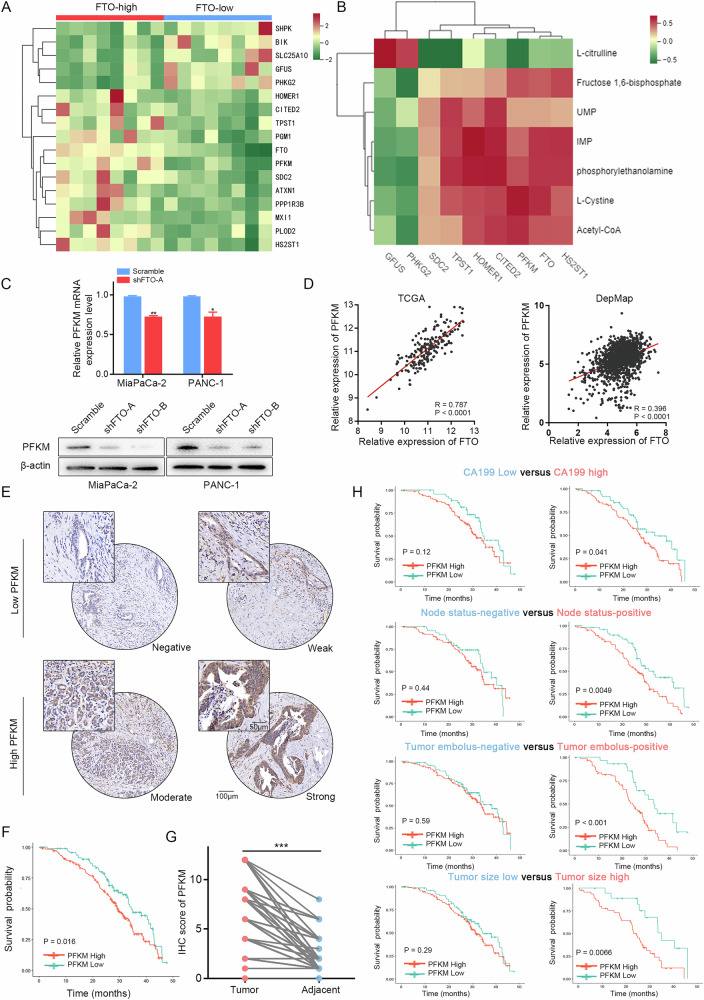
PFKM is correlated with poor prognosis in patients with PDAC. **A** Heatmap showing glycolysis-related genes between the FTO-high subgroup and the FTO-low subgroup. **B** Correlation analysis between glycolysis-related genes and differentially abundant metabolites based on the integrated analysis of transcriptomics and metabolomics. **C** FTO knockdown decreased PFKM mRNA and protein levels. **D** Correlations between FTO expression and PFKM expression in the DepMap and TCGA-PDAC databases. **E** Representative images are shown according to IHC staining for PFKM in TMAs. **F** Kaplan‒Meier analysis of OS in TMAs on the basis of PFKM expression. **G** IHC analysis of the PFKM staining score in PDAC and normal tissues. **H** Subgroup analyses of overall survival in TMAs based on PFKM level.

The list of DEGs in the panel associated with the functional enrichment of genes related to glycolysis was annotated via Metascape (Fig. 5A). Among the differentially expressed metabolic genes, PFKM is a second rate-limiting enzyme in glycolysis and plays a role in the formation of fructose-6-phosphate and fructose-1,6-diphosphate [15, 16]. Indeed, in the combined analyses of metabolomics and transcriptomic data, PFKM was positively correlated with carbohydrate metabolomics (Fig. 5B). Hence, PFKM was chosen as a candidate downstream effector gene of FTO that regulates glycolysis metabolism-mediated m^6^A modification. In the biological validation assay, PFKM mRNA and protein levels were confirmed to be downregulated in FTO-knockdown cells (Fig. 5C). Consistent with our earlier results, FTO and PFKM expression in the DepMap and TCGA databases was significantly positively correlated (*R* = 0.396, *P* < 0.0001; *R* = 0.787, *P* < 0.0001, respectively) (Fig. 5D). Furthermore, IHC staining was performed on tissue microarrays (TMAs) including tumor tissues from 278 patients (Cohort #3). Kaplan–Meier curves revealed that overall survival for subsets of patients with high PFKM expression was associated with poor prognosis in PDAC patients (*P* = 0.0016; Fig. 5E, F). Moreover, PFKM protein expression, as determined by the IHC score, was significantly greater in PDAC tissues than in adjacent tissues (Fig. 5G). Moreover, univariate and multivariate Cox regression analyses revealed that PFKM and other clinical characteristics, such as the presence of a tumor embolus, CA199 level and tumor size, were validated as independent prognostic factors in patients with PDAC (Table1). Subsequent survival analysis demonstrated that a high level of risk factors was a precondition for poor survival in patients with PDAC according to the PFKM (Fig. 5H).

**Table 1:** Univariate and multivariate Cox regression of overall survival for patients with PDAC.

	Univariate			Multivariate		
Characteristics	HR	95%CI	*P*	HR	95%CI	*P*
Age						
<60	1	0.720–1.427	0.937			
≥60	1.206					
Gender						
Female	1	0.719–1.348	0.923			
Male	0.985					
Perineural invasion (PNI)						
Negative	1	0.733–2.83	0.289			
Positive	1.441					
Lymph node status						
Negative	1	1.037–1.969	0.029			
Positive	1.429					
Tumor embolus						
Negative	1	1.271–2.412	<0.001	1	1.119–2.226	0.009
Positive	1.751			1.578		
CA199 level (u/ml)						
<200	1	1.081–2.048	0.015	1	1.024–1.975	0.036
≥200	1.488			1.422		
Tumor size (cm)						
<4	1	1.116–2.241	0.009	1	1.057–2.196	0.024
≥4	1.582			1.524		
PFKM expression						
Low	1	1.079–2.156	0.017	1	1.163–2.37	0.005
High	1.525			1.66		

Univariate *P* values were derived with log-rank test. Multivariate *P* values were derived with Cox regression analysis, *CI* indicates confidence interval; *HR* hazard ratio.

### FTO-induced restoration of C-Jun upregulated PFKM transcription

**Fig. 6:**
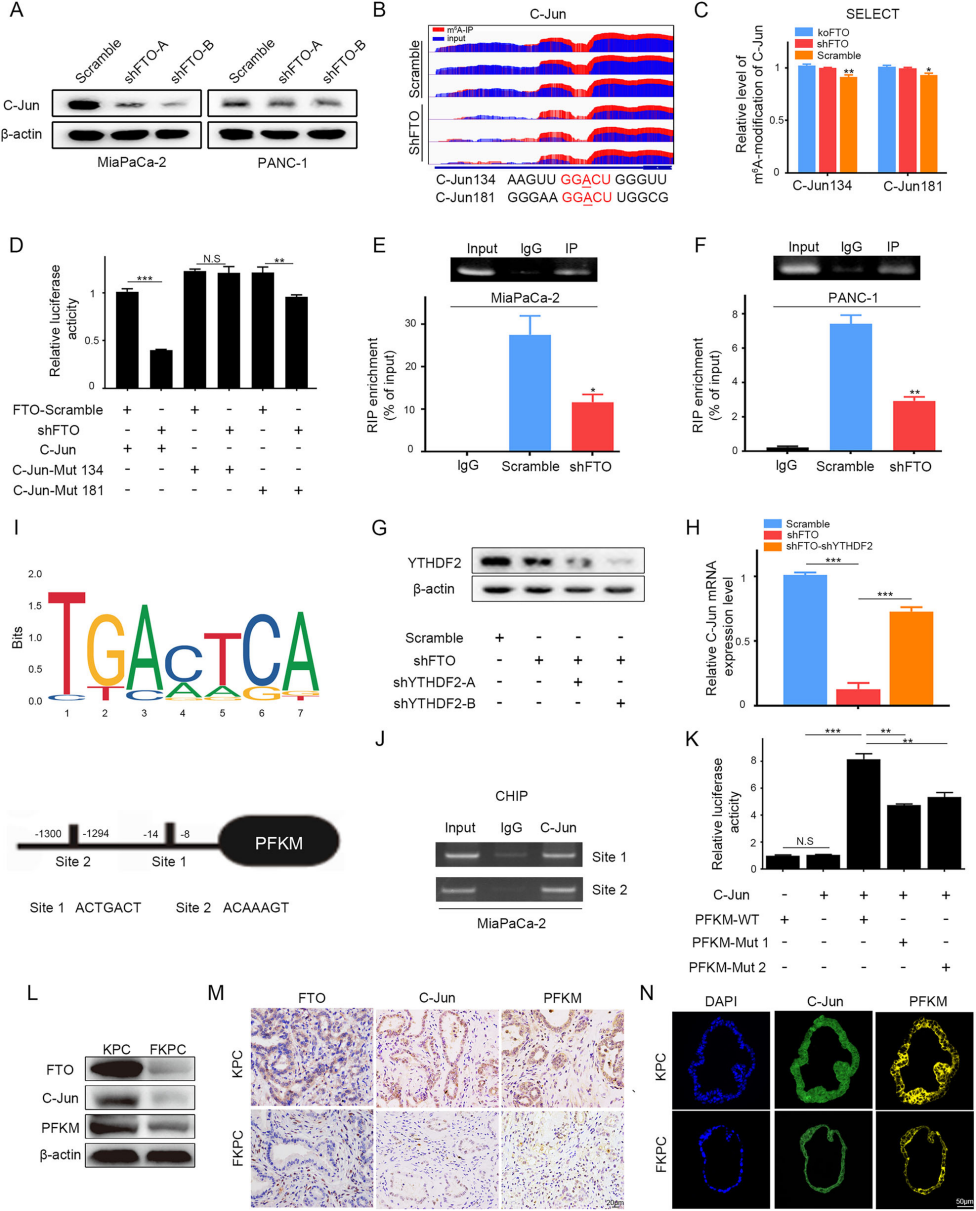
FTO activates PFKM transcription through C-Jun in a m^6^A-YTHDF2-dependent manner. **A** Immunoblotting of C-Jun and GAPDH in FTO-knockdown or scramble MiaPaCa-2 and PANC-1 cells. **B** IGV plots showing m^6^A modification of C-Jun mRNA during FTO attenuation and scramble treatment. **C** qPCR revealed that FTO significantly decreased m^6^A modification levels at 2 sites. **D** Relative luciferase activity of the wild-type and mutant PDGFC 3ʹ UTR reporter vectors. **E**, **F** C-Jun mRNA was immunoprecipitated by YTHDF2, and the transcript level was assessed via qRT‒PCR. **G** YTHDF2 protein levels in MiaPaCa-2 cells transfected with scramble, sh-FTO, or cotransfected with sh-FTO or sh-YTHDF2 were detected by immunoblotting. **H** qRT‒PCR analysis of C-Jun mRNA levels in the presence of scramble or shYTHDF2 in the absence or presence of FTO knockdown. **I** The C-Jun-binding motif and sites enriched in the PFKM promoter were predicted via the JASPAR website. **J** ChIP analysis of MiaPaCa-2 and PANC-1 cells revealed the binding of C-Jun and 2 sites in the PFKM promoter. **K** A luciferase reporter assay was performed for the wild-type or mutant sequence of C-Jun binding sites to verify the activity of sites 1 and 2 in PFKM; IB (**L**), IHC (**M**) and IF (**N**) images showing C-Jun and PFKM expression in spontaneous PDAC samples from the indicated KPC/FKPC mice.

The bZIP family of transcription factors, including C-Jun, C/EBPb and JunB, is well known to be associated with the transcriptional activation of genes encoding glycolysis enzymes [17]. We concluded that glycolysis inhibition upon FTO knockdown might be due to the downregulated expression of these transcription factors. Indeed, we found that C-Jun was downregulated at the RNA and protein levels when FTO was knocked down (Fig. 6A). To further validate that C-Jun was the downstream substrate and demethylation catalyzed by FTO. MiaPaCa-2 cells were treated with FB23-2 for 24 h. The m^6^A dot blot results showed that inhibiting the activity of the FTO enzyme could enhance the m^6^A modification in PDAC cells. Western blot assays showed that C-Jun level decreased with the increase of m^6^A level (Supplementary Fig. S1B). We next used Integrative Genomics Viewer (IGV) software according to the MeRIP-seq data to detect C-Jun m^6^A peak enrichment, which was markedly increased around the 3’ UTR in FTO-knockdown cells (Fig. 6B). Putative m^6^A sites within the C-Jun mRNA 3’UTR were predicted using the online tools SRAMP (http://www.cuilab.cn/sramp) and RMBase (https://rna.sysu.edu.cn/rmbase/m^6^Amod.php), both trained on high-confidence human m^6^A-seq datasets. To assess the m^6^A level of the methylated sites of C-Jun, we utilized a recently developed technique known as SELECT, which is based on single-base elongation and ligation qPCR amplification. The results revealed that FTO significantly increased m^6^A modification levels at these two sites in FTO in the RNA lysates of 293 T cells (Fig. 6C). To explore the relationship between FTO and the m^6^A modification sites of C-Jun, we replaced the adenosine base in the m^6^A consensus sequence with thymine at the two potential m^6^A sites of C-Jun mRNA. We subsequently individually cloned these genes into a dual-luciferase reporter construct and conducted luciferase reporter gene assays. As shown in Fig. 6D, FTO knockdown effectively inhibited the luciferase activity of C-Jun, and site-directed mutagenesis (C-Jun-Mut134) reversed this effect. It is generally believed that specific reader proteins facilitate m^6^A-dependent functions and mediate complex mechanisms of m^6^A regulation [18]. YTHDF2 recruits the CCR4-NOT complex deadenylation mediator and ultimately contributes to the degradation of m^6^A-modified RNA [19]. Consequently, on the basis of the mechanism by which YTHDF2 functions, we hypothesized that C-Jun mRNA could interact with YTHDF2. RIP assays further confirmed the specific interaction between YTHDF2 and C-Jun. As shown in Fig. 6E, F, YTHDF2 enrichment at C-Jun transcripts was significantly reduced when FTO was stably knocked down in PDAC cells. Moreover, we also knocked down YTHDF2 in FTO-knockdown PDAC cells and detected that C-Jun expression partially recovered (Fig. 6G, H). Our data indicate that the C-Jun mRNA level is regulated by FTO in a m^6^A-YTHDF2-dependent manner. Using the JASPAR genomic platform, two C-Jun-binding domains were recognized in the PFKM promoter (Fig. 6I). Then, ChIP analysis was performed to elucidate the DNA-binding interactions of transcription factors. As shown in Fig. 6J, C-Jun was demonstrated to bind to both PFKM sites in MiaPaCa-2 cells. We cloned a fragment of the PFKM promoter into a luciferase reporter vector. Further validation via a luciferase assay revealed that C-Jun could stimulate the expression of wild-type PFKM promoter activity, whereas mutations in C-Jun-binding site 1 or site 2 inhibited the interaction (Fig. 6K). To further validate the association of FTO expression with C-Jun and PFKM, we analyzed the expression patterns in KPC/FKPC mice. FTO, C-Jun and PFKM were individually detected using WB analysis in transgenic mouse primary tumor cells (Fig. 6L). IHC and IF staining also revealed that FTO deletion decreased C-Jun and PFKM expression in tumor tissues and MDO (Fig. 6M, N).

### FTO-induced metastasis depends on the activation of glycolysis in PDAC

**Fig. 7:**
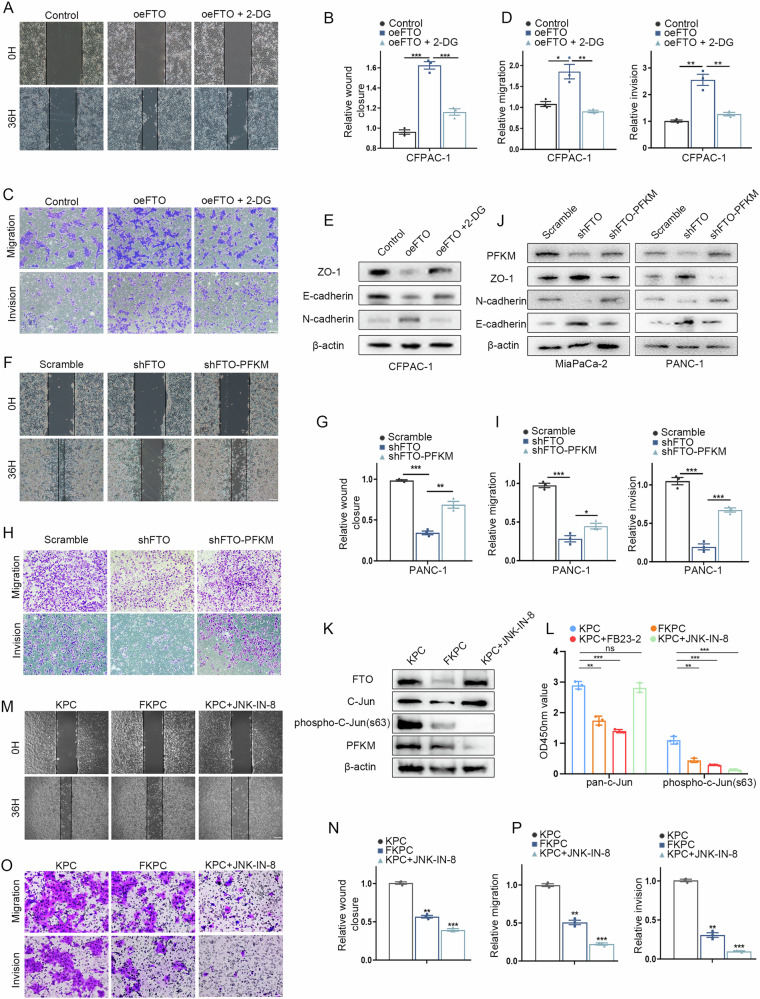
PFKM plays a key role in FTO-modulated PDAC metastasis. **A**–**D** Wound healing and Transwell assays showing the migratory capacity of CFPAC-1 cells treated with empty vector or FTO and treated with or without 2-DG. **E** EMT-related genes in cells overexpressing FTO with or without 2-DG were detected by immunoblotting. **F**–**I** Wound healing and Transwell assays were performed in FTO-knockdown PANC-1 cells with or without additional PFKM expression. **J** The protein levels of EMT-related genes were determined via Western blotting in MiaPaCa-2 and PANC-1 cells with PFKM overexpression with or without FTO knockdown. **K** KPC/FKPC primary cells were treated with the JNK inhibitor JNK-IN-8, and changes in the indicated targets were assessed by immunoblotting. **L** ELISA analysis of phospho-C-Jun (Ser63) and total C-Jun levels in the supernatants of KPC/FKPC primary cells and treated with JNK-IN-8 or FB23-2. **M**–**P** Wound healing and transwell assays showing the migratory capacity of KPC/FKPC primary cells and treatment with JNK-IN-8.

**Fig. 8:**
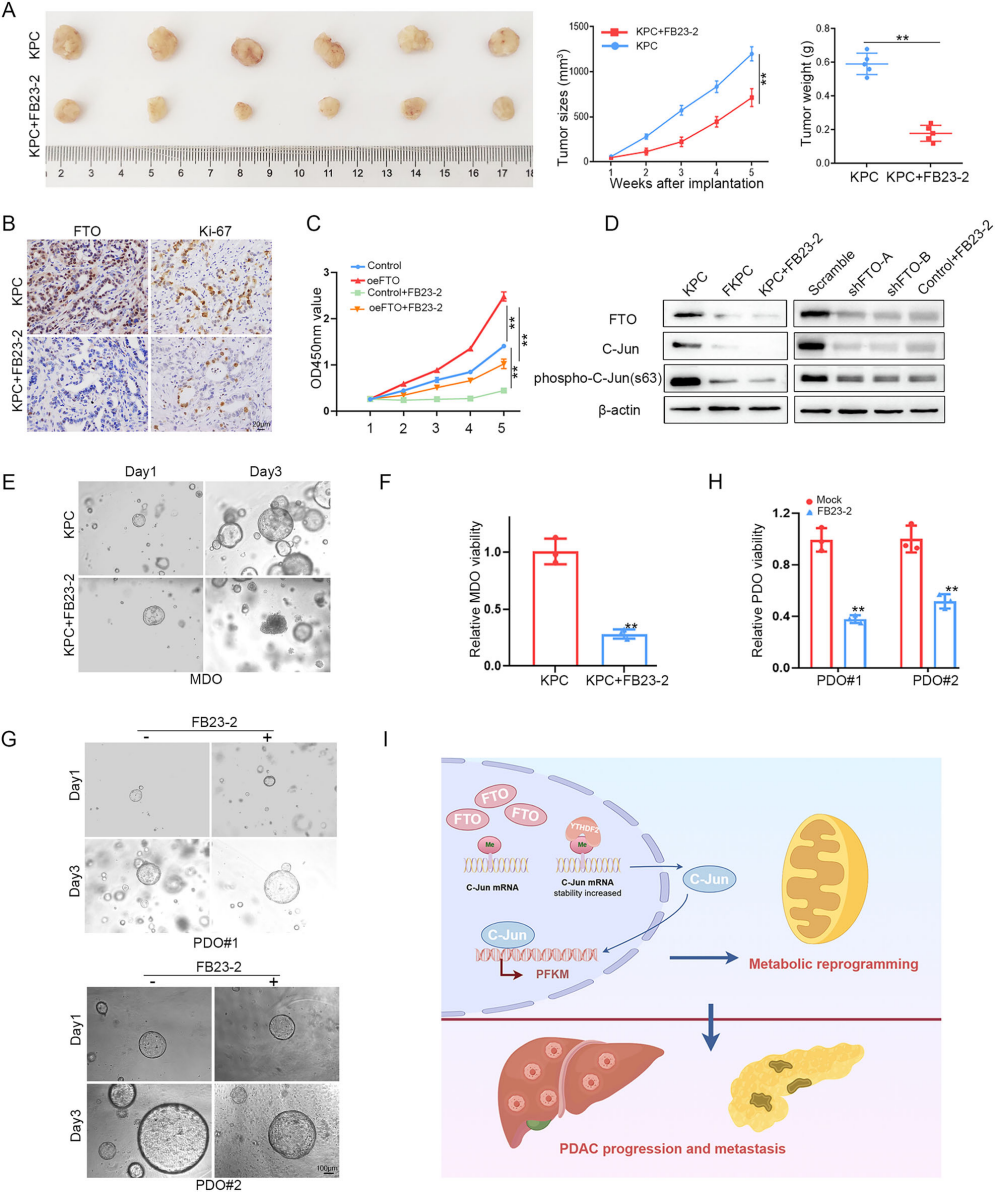
Targeting FTO inhibited pancreatic tumor growth. **A** Representative image of subcutaneous tumors formed from KPC primary cells and FB23-2. Tumor volumes and weights were measured after excision from the stated nude mouse groups. **B** FTO and Ki67 protein expression levels in xenograft tumors were measured via IHC staining. **C** Phospho-C-Jun (Ser63) and C-Jun protein levels were determined via WB in KPC/FKPC primary cells or upon treatment with FB23-2 and after FTO OE or KD in MiaPaCa-2 and CFPAC-1 cells. **D** Cell proliferation ability was detected using the CCK-8 assay in FTO-overexpressing cells and FB23-2-overexpressing cells. **E**–**H** MDO and PDO 3D organoids were imaged under a microscope, and viability was determined via WST-1 assays. **I** Schematic diagram of the function and mechanism of FTO/C-Jun/PFKM in metabolic reprogramming and metastasis in PDAC.

FTO, a m^6^A regulator, is associated with PDAC metastasis and EMT pathway activation. Our data confirmed that FTO upregulates PFKM and enhances glycolytic metabolism. Indeed, we aimed to investigate whether the FTO/C-Jun/PFKM axis contributes to tumorigenesis and metastasis by modulating glycolysis. In gain-of-function assays, FTO overexpression significantly increased cell migration and invasion abilities. To test whether the capacity for glycolysis is necessary for PDAC cell migration, we treated PDAC cells with an inhibitor of glycolysis (2-DG) to inhibit their glycolytic capacity and observed significantly blocked cell migration and invasion abilities induced by FTO as well as the expression of EMT-related genes (Fig. 7A–E). These data indicate that FTO may act as a driver gene to promote PDAC progression by regulating glucose metabolism. To explore whether PFKM downregulation accounts for the decrease in migration caused by FTO knockdown, we re-expressed PFKM in FTO-knockdown cells. The results of the wound healing and transwell assays revealed that FTO knockdown remarkably weakened the ability, but the effects were restored by the increased expression of PFKM (Fig. 7F–I and Supplementary Fig. S1E–F). In MiaPaCa-2 and PANC-1 cells with FTO knockdown, there was an increase in E-cadherin and ZO-1, and a decrease in N-cadherin. In line with the findings of the above study, the reduction E-cadherin, N-cadherin and ZO-1 expression was rescued by overexpression of PFKM, revealing that FTO promotes PDAC cell migration capabilities partially via PFKM expression (Fig. 7J). To verify the regulatory mechanism of the FTO/C-Jun/PFKM axis, we further executed C-Jun blocking experiments in KPC/FKPC primary tumor cells. JNK-IN-8, which is a specific inhibitor of JNK, inhibits the phosphorylation of the downstream molecule C-Jun [20, 21]. Decreased C-Jun phosphorylation and PFKM expression were observed following JNK-IN-8 and FB23-2 treatment, as detected by ELISA and Western blot (Fig. 7K, L). Moreover, the absence of FTO significantly decreased the migration and invasion abilities of KPC cells, whereas comparable effects were observed after JNK-IN-8 treatment (Fig. 7M‒P). To gain more insight into the potential impact of targeting FTO, we employed a PDAC xenograft model with FTO inhibitor treatment to explore the effect of this potential therapy against pancreatic cancer. For the PDAC subcutaneous tumorigenesis model, the mice were intraperitoneally administered the FTO inhibitor FB23-2 or DMSO. Tumor volume and weight were measured during the therapeutic period, and the data consistently suggested that FB23-2 treatment significantly inhibited tumor progression (Fig. 8A). Additionally, IHC staining revealed dramatically lower FTO and Ki-67 expression in the inhibitor-treated group than in the mock-treated group (Fig. 8B). The CCK8 assay results revealed that FTO overexpression increased PDAC cell proliferation and that FB23-2 visibly inhibited viability in vitro (Fig. 8C). We next wanted to explore whether FB23-2 performs its biological function through the FTO/C-Jun/PFKM axis. We found that the phosphorylation of C-Jun in the FTO-overexpressing group was significantly greater than that in the control group, whereas FB23-2 reversed these effects (Fig. 8D). Moreover, we established an individual PDO model from resected primary human PDAC tissues from FUSCC to evaluate the therapeutic effect of FB23-2. Compared with mock control organoids, FB23-2-treated organoids significantly suppressed the growth and activity of PDOs and MDOs (Fig. 8E–H). Therefore, our results indicate that targeting the FTO/C-Jun/PFKM axis is a potentially effective intervention strategy for treating PDAC.

## Discussion

Despite considerable progress in understanding genetic diversity, limited progress has been made in identifying the molecular changes associated with the highly metastatic PDAC phenotype [22, 23]. Recent studies have revealed that m^6^A methylation is an important mechanism involved in pancreatic carcinogenesis [24]. In this study, using LC‒MS/MS and RNA-seq, we demonstrated that the expression of the m^6^A demethylase FTO is highly increased in m^6^A-low subgroup patients with increased FDG uptake. Combined transcriptomics and metabolomics data reveal new functional modules of FTO associated with glucose metabolism and metastasis in PDAC.

RNA m^6^A modifications have been shown to participate in numerous biological functions and cancer therapeutic responses [25]. Indeed, an extensive body of literature indicates that m^6^A regulators are strongly correlated with cancer pathway activation and with prognostically relevant tumor subtypes [6, 26]. Since FTO was recognized as the first m^6^A mRNA demethylase, an increasing number of reports have connected m^6^A demethylation with a range of biological processes [27–29]. However, the potential involvement of m^6^A modification in metabolic processes in PDAC remains unknown. In our study, transgenic mice, organoids and a metastatic xenograft model were used to investigate the role of FTO in cancer biology. Compared with KPC depletion, FTO depletion prolonged long-term survival in a spontaneous mouse model and hypometabolism, as shown by PET imaging.

The combination of metabolomics and transcriptomic data demonstrated that glycolysis pathways were enriched, and PFKM was chosen as a candidate target of FTO. We further showed that FTO dominates glycolysis by preserving C-Jun expression in a m^6^A-YTHDF2-dependent manner. Attenuating FTO expression decreases C-Jun accumulation and subsequently reduces the transcription of PFKM in PDAC cells, which subsequently abolishes the metabolic supply for migration. A recent study demonstrated that FTO stimulates the glycolytic activity of tumor cells, thus modulating CD8^+^ T-cell function and promoting melanoma development [9]. However, in papillary thyroid cancer, FTO acts as a tumor suppressor to inhibit APOE expression and hinder glycolysis via the IL-6/JAK2/STAT3 signaling pathway [30]. These results indicate the heterogeneity of FTO functions in different cancer types. Hence, defining the distinct demethylation substrate RNA molecules of FTO across multiple tumor types will be informative in the future.

Increased metabolism during glycolysis is the major consequence of increased activation through the rapid reformation of ATP, which is required for oncogenic processes [31]. Tumor cells with elevated levels of glycolytic activity (Warburg effect) facilitate the biology of tumor progression [32]. PFKM is a rate-limiting enzyme in glycolysis that catalyzes fructose 6-phosphate and ATP. Prior studies revealed that the PFKM affects the rate of cellular glycolysis, thus contributing to the availability of extracellular nutrients and stimulating growth signals [16, 33]. In ovarian cancer and hepatocellular carcinoma, PFKM expression is positively correlated with poor survival and progression [34, 35]. However, the mechanisms underlying the increased PFKM expression in pancreatic cancer has not been clearly demonstrated. Hence, we performed survival analysis of the PFKM on our TMAs and revealed that it was associated with poor outcomes in patients with PDAC. Subsequent survival analysis demonstrated that a high level of markers associated with metastatic risk was a precondition for the ability of PFKM to predict poor survival in patients with PDAC. This finding also suggests that PFKM is potentially associated with the metastatic potential of pancreatic cancer. We then attempted to identify the biological mechanisms underlying the observed associations. Rescue experiments conducted with 2-DG and JNK-IN-8 revealed that FTO-enhanced glycolysis further promoted the maintenance of cell invasion via the YTHDF2/C-Jun/PFKM pathway. PFKM overexpression in FTO-knockdown cells also rescued the migratory capability resulting from reduced FTO expression. Our results highlight a novel mechanism by which FTO promotes PDAC tumorigenesis by modulating glycolysis levels. Blocking the glycolytic pathway is a new approach for cancer treatment because PDAC involves high levels of glycolysis to meet its metabolic requirements. PDO and MDO models include various subsets within tumors, preserve their histological characteristics and remain stable across passages [36]. These models replicate the genetic/epigenetic genetic features of the original tumor tissues, as well as their responses to cancer therapies [37]. Translational studies involving organoid models and xenograft tumor models revealed that the use of FTO inhibitors significantly suppressed PDAC growth. Considering the rapid increase in interest in RNA-based therapies, targeting the m^6^A-dependent FTO/C-Jun/PFKM glycolysis regulatory axis may be essential for the prevention and treatment of PDAC.

Overall, we demonstrated that m^6^A modification is partially correlated with glycolytic activation in pancreatic cancer. The findings showed that FTO preserved C-Jun expression through m^6^A-YTHDF2-dependent modifications. These changes result in increased PFKM transcription as well as glycolysis activation and subsequently abolish the metabolic barrier for migration. m^6^A can be used as a supplementary layer of genetic regulation for inducing metabolic reprogramming, which could facilitate the discovery of efficient therapeutic strategies potentially against PDAC.

### Reporting summary

Further information on research design is available in the Nature Portfolio Reporting Summary linked to this article.

## Supplementary information

Reporting Summary

Table. S1-3

supplement WB

Supplementary materials

## Data Availability

The data are available from the corresponding author upon reasonable request.
