# Supplementary material for: m6A demethylase FTO drives pancreatic ductal adenocarcinoma tumorigenesis and metastasis through remodeling PFKM mediated glycolysis
Source: Cell Death Dis. 2025 Nov 3;16(1):784. doi: 10.1038/s41419-025-08049-2 (PMC12583531; doi:10.1038/s41419-025-08049-2)
Supplement: Supplementary file 2 — Table. S1-3 [file 41419_2025_8049_MOESM2_ESM.docx]

| Sample | Gender | Age | Location | Sample type |
| --- | --- | --- | --- | --- |
| FUS101 | Female | 43 | Body-tail | Frozen |
| FUS102 | Male | 65 | Body-tail | Frozen |
| FUS103 | Male | 62 | Body-tail | Frozen |
| FUS104 | Male | 61 | Body-tail | Frozen |
| FUS105 | Female | 81 | Head | Frozen |
| FUS106 | Male | 61 | Body-tail | Frozen |
| FUS107 | Male | 58 | Head | Frozen |
| FUS108 | Female | 61 | Body-tail | Frozen |
| FUS109 | Female | 57 | Head | Frozen |
| FUS110 | Female | 52 | Body-tail | Frozen |
| FUS111 | Female | 54 | Body-tail | Frozen |
| FUS112 | Female | 77 | Body-tail | Frozen |
| FUS113 | Male | 70 | Body-tail | Frozen |
| FUS114 | Male | 67 | Body-tail | Frozen |
| FUS115 | Male | 51 | Head | Frozen |
| FUS116 | Male | 44 | Head | Frozen |

Table S1. Characteristics for patients of Cohort-1.

| Sample | Gender | Age | Location | Sample type |
| --- | --- | --- | --- | --- |
| FUS201 | Male | 64 | Head | Frozen |
| FUS202 | Male | 59 | Body-tail | Frozen |
| FUS203 | Female | 69 | Body-tail | Frozen |
| FUS204 | Male | 67 | Body-tail | Frozen |
| FUS205 | Male | 55 | Body-tail | Frozen |
| FUS206 | Male | 59 | Head | Frozen |
| FUS207 | Male | 63 | Body-tail | Frozen |
| FUS208 | Male | 68 | Head | Frozen |
| FUS209 | Male | 41 | Head | Frozen |
| FUS210 | Female | 66 | Body-tail | Frozen |
| FUS211 | Female | 71 | Head | Frozen |
| FUS212 | Female | 74 | Body-tail | Frozen |
| FUS213 | Male | 74 | Body-tail | Frozen |
| FUS214 | Female | 66 | Body-tail | Frozen |
| FUS215 | Female | 53 | Head | Frozen |
| FUS216 | Male | 65 | Head | Frozen |
| FUS218 | Male | 70 | Head | Frozen |
| FUS217 | Male | 77 | Body-tail | Frozen |

Table S2. Characteristics for patients of Cohort-2.

|  | PFKM-High | PFKM-Low |
| --- | --- | --- |
| Age(year) |  |  |
| <=60 | 67 | 34 |
| >60 | 114 | 63 |
| Gender |  |  |
| Male | 107 | 56 |
| Female | 74 | 41 |
| Tumor location |  |  |
| Head | 92 | 32 |
| Body/tail | 89 | 65 |

Table S3. Characteristics for patients of Cohort-3.
