# Supplementary figures and images for: m6A demethylase FTO drives pancreatic ductal adenocarcinoma tumorigenesis and metastasis through remodeling PFKM mediated glycolysis
Source: Cell Death Dis. 2025 Nov 3;16(1):784. doi: 10.1038/s41419-025-08049-2 (PMC12583531; doi:10.1038/s41419-025-08049-2)

Fig.2

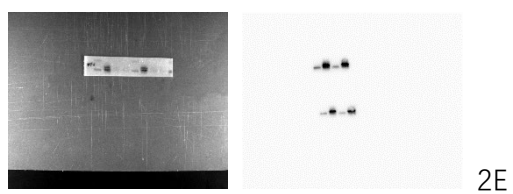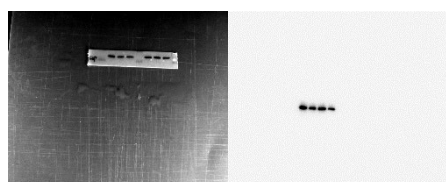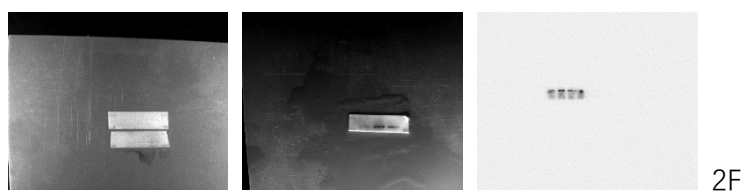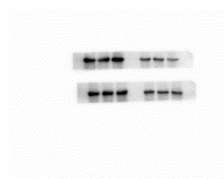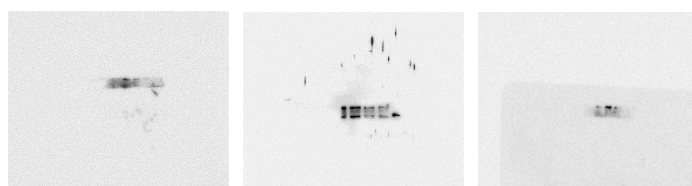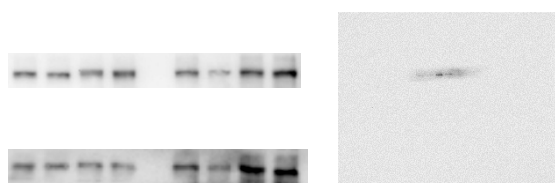

Fig.5

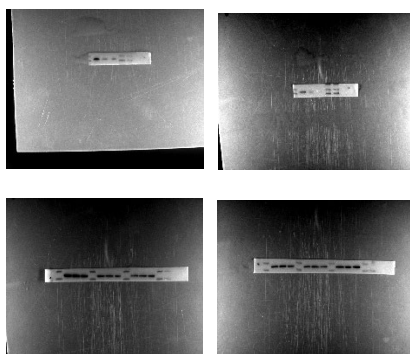

Fig.6

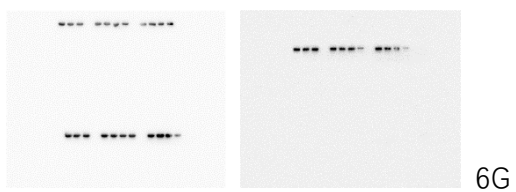

6G

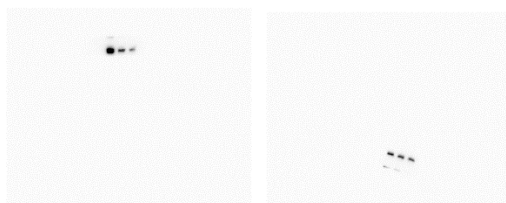

6A

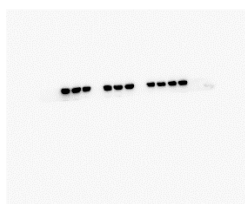

6A

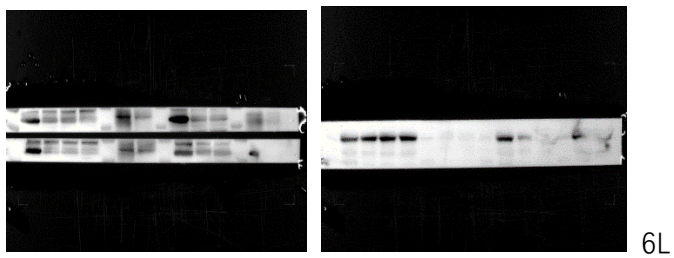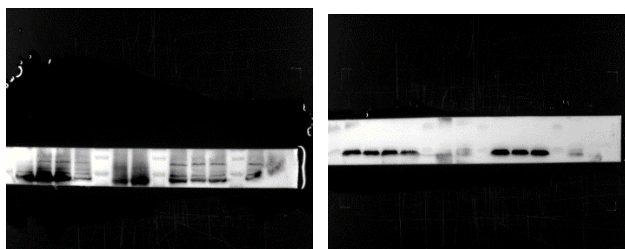

Fig.7

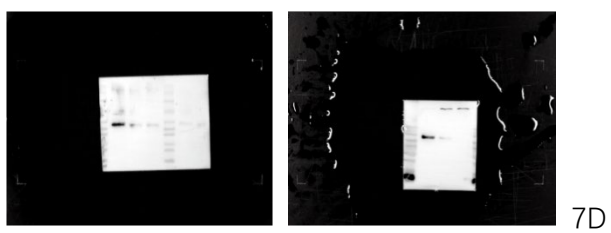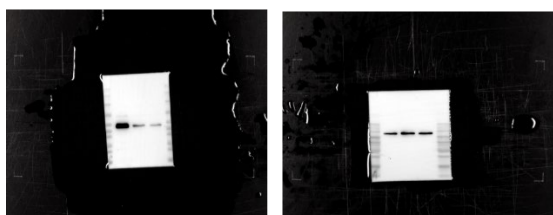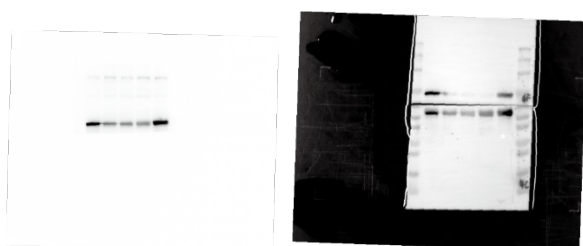

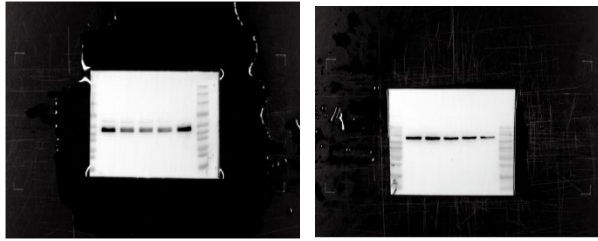

Fig.S1

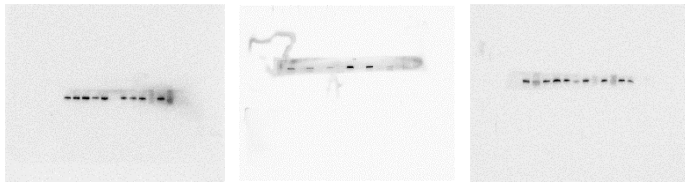

S1A

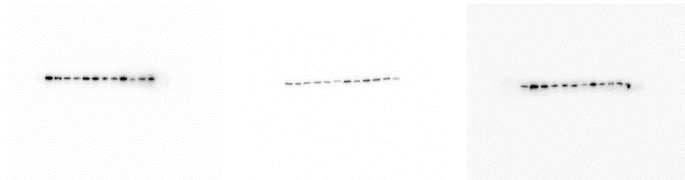

Fig.S3

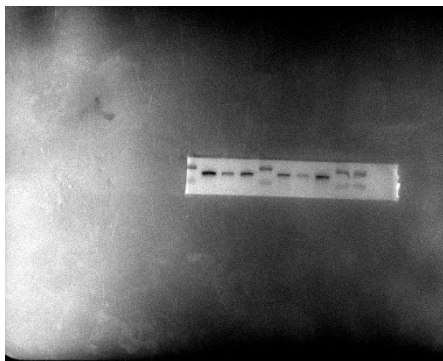

S3J

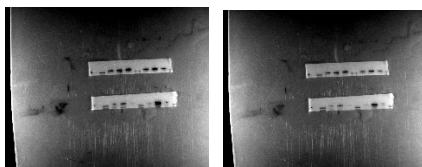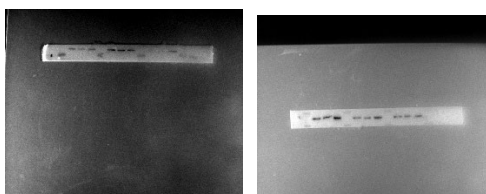

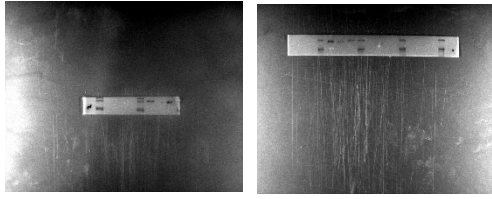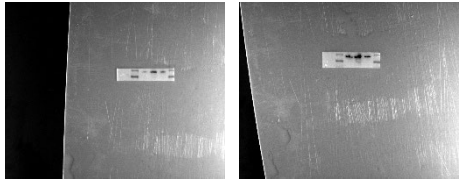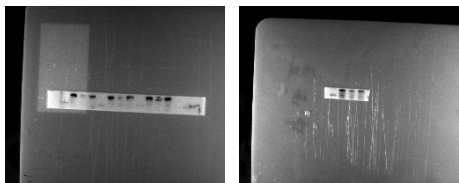

S3E

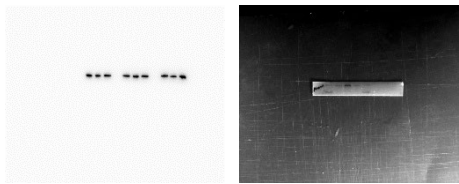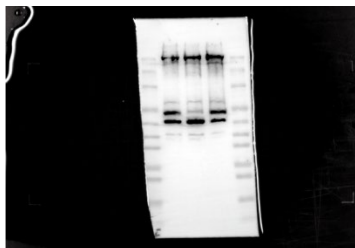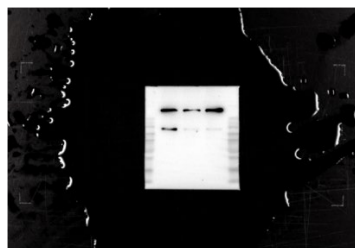

S3K

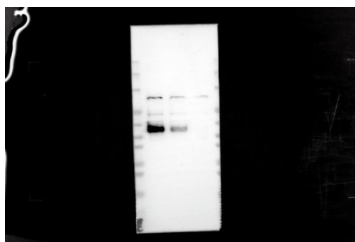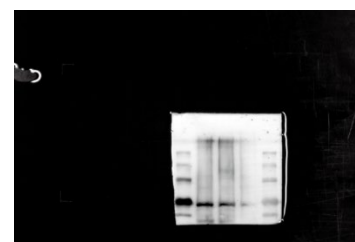

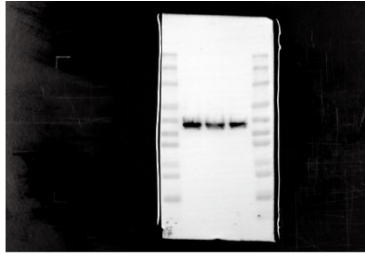

Supplement: Supplementary file 3 — supplement WB [file 41419_2025_8049_MOESM3_ESM.pdf]
