# Supplementary material for: m6A demethylase FTO drives pancreatic ductal adenocarcinoma tumorigenesis and metastasis through remodeling PFKM mediated glycolysis
Source: Cell Death Dis. 2025 Nov 3;16(1):784. doi: 10.1038/s41419-025-08049-2 (PMC12583531; doi:10.1038/s41419-025-08049-2)
Supplement: Supplementary file 4 — Supplementary materials [file 41419_2025_8049_MOESM4_ESM.docx]

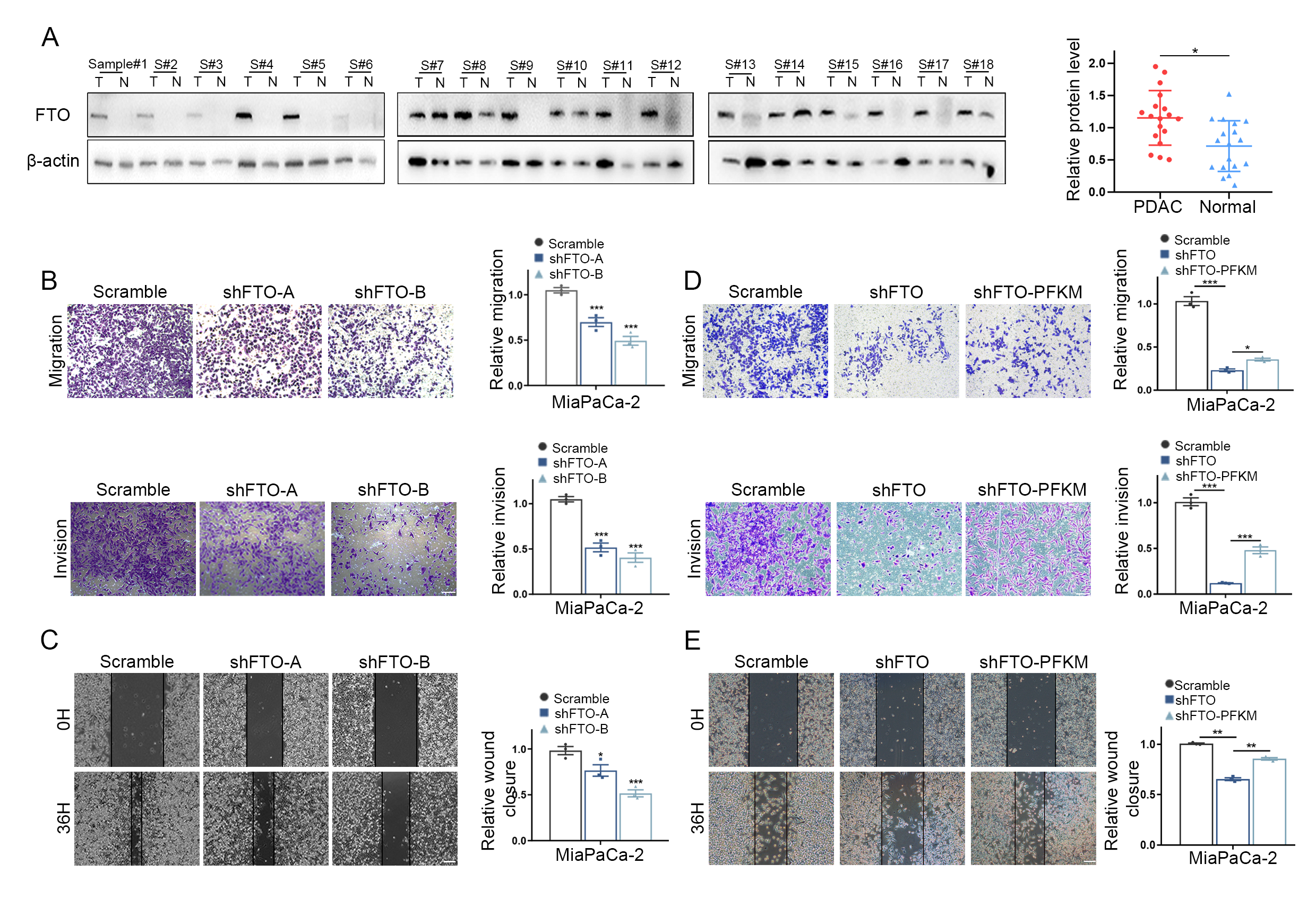


**Fig. S1** (A) Western blotting verified the increased FTO levels in PDAC samples (T) compared with normal tissues (N) (Cohort #2). (B-C) FTO knockdown significantly decreased the migratory capacity of MiaPaCa-2 cells. (D-E) Wound healing and Transwell assays were performed in FTO-knockdown MiaPaCa-2 cells with or without further expression of PFKM.


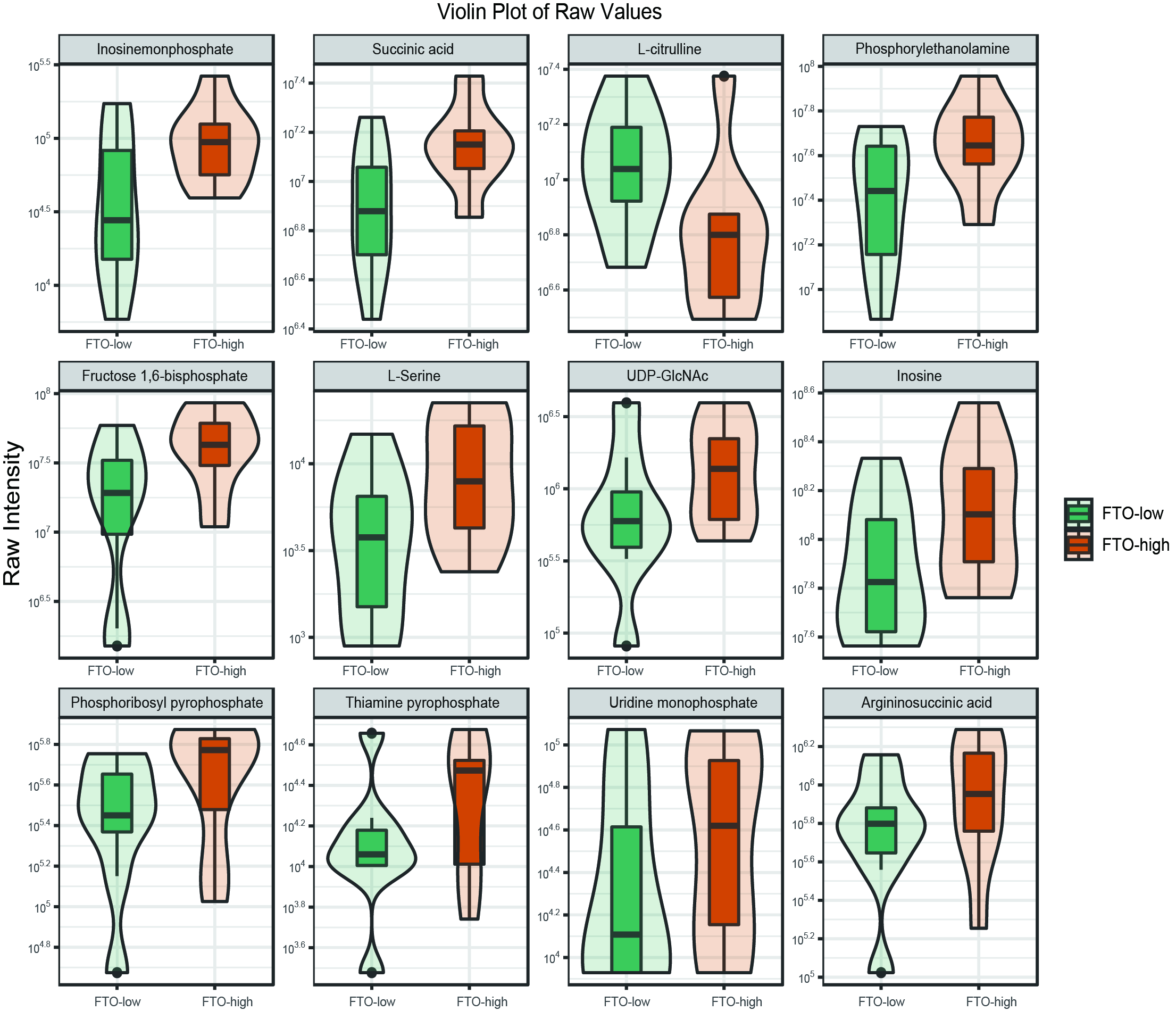


**Fig. S2** Violin plots indicating the density distributions of 12 types of differentially abundant metabolites between the two groups.


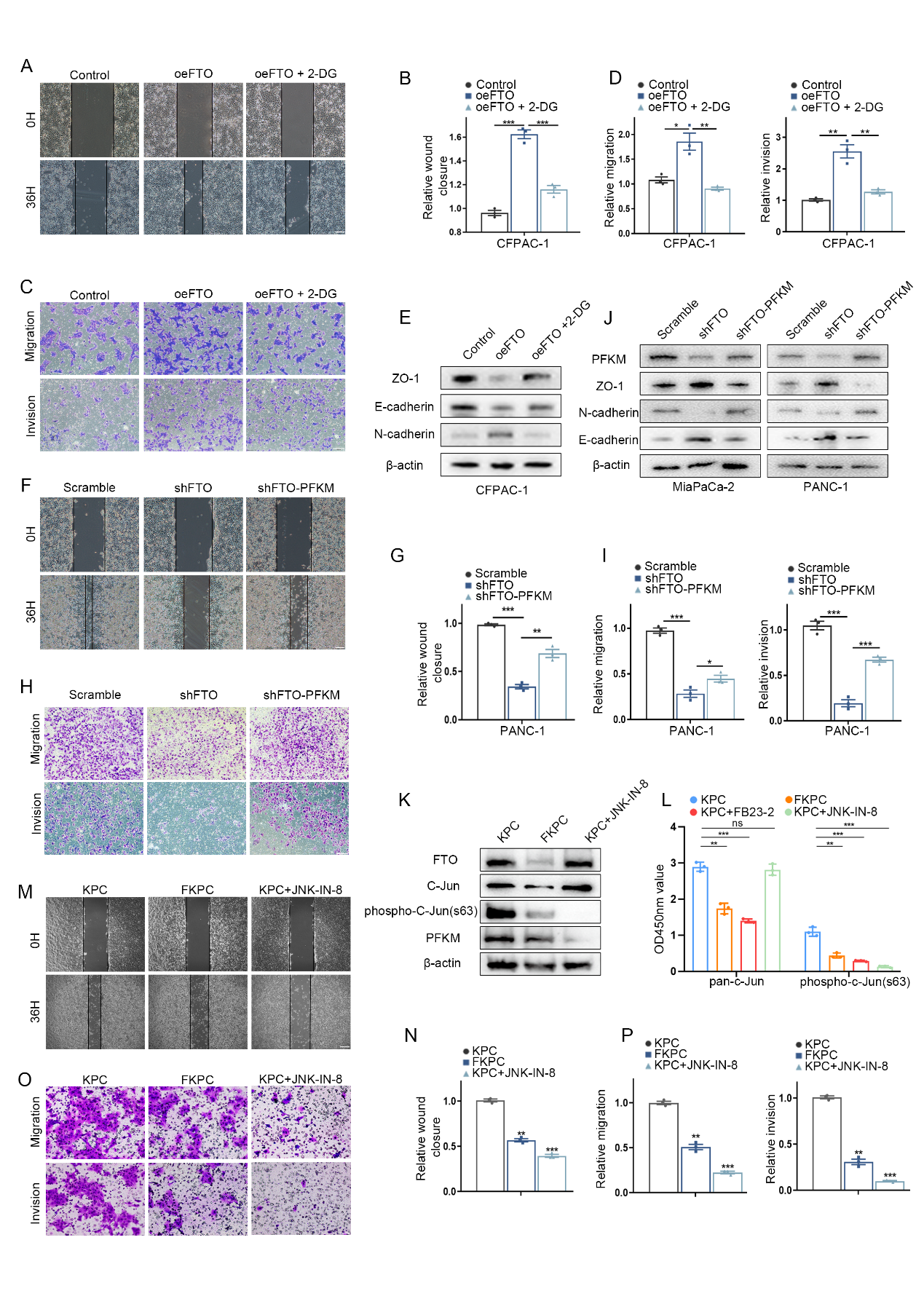


**Fig. S3 PFKM plays a key role in FTO-modulated PDAC metastasis.** (A-D) Wound healing and Transwell assays showing the migratory capacity of CFPAC-1 cells treated with empty vector or FTO and treated with or without 2-DG. (E) EMT-related genes in cells overexpressing FTO with or without 2-DG were detected by immunoblotting. (F-I) Wound healing and Transwell assays were performed in FTO-knockdown PANC-1 cells with or without additional PFKM expression. (J) The protein levels of EMT-related genes were determined via Western blotting in MiaPaCa-2 and PANC-1 cells with PFKM overexpression with or without FTO knockdown. (K) KPC/FKPC primary cells were treated with the JNK inhibitor JNK-IN-8, and changes in the indicated targets were assessed by immunoblotting. (L) ELISA analysis of phospho-C-Jun (Ser63) and total C-Jun levels in the supernatants of KPC/FKPC primary cells and treated with JNK-IN-8 or FB23-2. (M-P) Wound healing and transwell assays showing the migratory capacity of KPC/FKPC primary cells and treatment with JNK-IN-8.
